# Nitrogenous Compounds from the Antarctic Fungus *Pseudogymnoascus* sp. HSX2#-11

**DOI:** 10.3390/molecules26092636

**Published:** 2021-04-30

**Authors:** Ting Shi, Li Zheng, Xiang-Qian Li, Jia-Jia Dai, Yi-Ting Zhang, Yan-Yan Yu, Wen-Peng Hu, Da-Yong Shi

**Affiliations:** 1 State Key Laboratory of Microbial Technology, Institute of Microbial Technology, Shandong University, Qingdao 266200, China; shiting_jia@126.com (T.S.); lixiangqian@sdu.edu.cn (X.-Q.L.); daijiajia@sdu.edu.cn (J.-J.D.); or 201700140016@sdu.edu.cn (Y.-T.Z.); yuyanyan@sdu.edu.cn (Y.-Y.Y.); wenpeng19961202@163.com (W.-P.H.); 2 Key Laboratory of Marine Eco-Environmental Science and Technology, First Institute of Oceanography, Ministry of Natural Resources, Qingdao 266061, China; zhengli@fio.org.cn; 3 Laboratory for Marine Ecology and Environmental Science, Qingdao Pilot National Laboratory for Marine Science and Technology, Qingdao 266071, China; 4 Laboratory for Marine Drugs and Bioproducts of Qingdao National Laboratory for Marine Science and Technology, Qingdao 266071, China

**Keywords:** Antarctic fungus, *Pseudogymnoascus* sp., secondary metabolites, nitrogenous compounds

## Abstract

The species *Pseudogymnoascus* is known as a psychrophilic pathogenic fungus which is ubiquitously distributed in Antarctica. While the studies of its secondary metabolites are infrequent. Systematic research of the metabolites of the Antarctic fungus *Pseudogymnoascus* sp. HSX2#-11 led to the isolation of one new pyridine derivative, 4-(2-methoxycarbonyl-ethyl)-pyridine-2-carboxylic acid methyl ester (**1**), together with one pyrimidine, thymine (**2**), and eight diketopiperazines, *cyclo*-(dehydroAla-l-Val) (**3**), *cyclo*-(dehydroAla-l-Ile) (**4**), *cyclo*-(dehydroAla-l-Leu) (**5**), *cyclo*-(dehydroAla-l-Phe) (**6**), *cyclo*-(l-Val-l-Phe) (**7**), *cyclo*-(l-Leu-l-Phe) (**8**), *cyclo*-(l-Trp-l-Ile) (**9**) and *cyclo*-(l-Trp-l-Phe) (**10**). The structures of these compounds were established by extensive spectroscopic investigation, as well as by detailed comparison with literature data. This is the first report to discover pyridine, pyrimidine and diketopiperazines from the genus of *Pseudogymnoascus*.

## 1. Introduction

**Figure 1:**
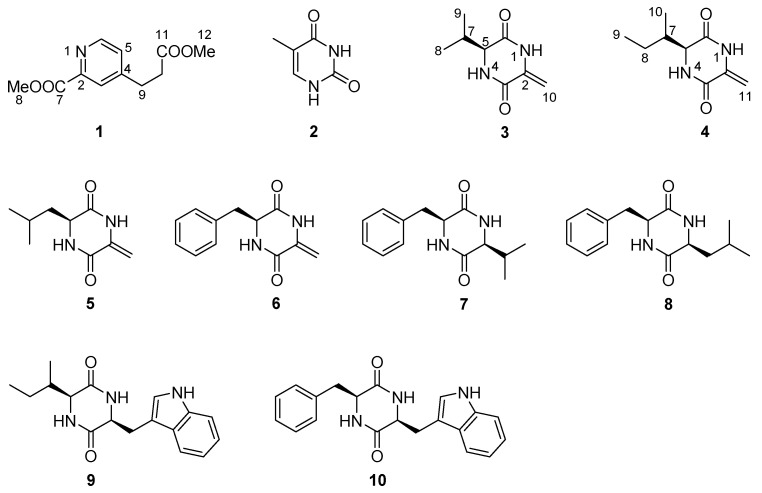
Structures of compounds **1**–**10**.

Nitrogenous compounds represent one of the most momentous family of secondary metabolites which are widely distributed in different biological sources [1]. They have been proved to exhibit various biological activities including cytotoxic, anti-inflammatory, antimicrobial activities and so on [1,2,3,4]. For example, pegaharine D, a β-carboline alkaloid isolated from the seeds of *Peganum harmala* exhibited strong antiviral activity against herpes simplex virus-2 [5]. For another example, asperversiamides A–C, a kind of cycloheptapeptide, showed potent inhibitory activity against *Mycobacterium marinum* [6]. Antarctica as the southernmost point of the earth, has the most hostile environment including cold, dry climate and low level of nutrition [7]. Microbes, especially fungi, have been proved to have the potential capacity to produce abundant novel compounds to adapt the extreme habitat. There were more and more bioactive natural products with novel structures have been discovered from Antarctic fungi [8,9,10,11]. The species *Pseudogymnoascus* is known as a psychrophilic pathogenic fungus with a ubiquitous distribution in Antarctica. While the rare research about its secondary metabolites suggested the potentials to discover interesting compounds [12]. *Pseudogymnoascus* sp. HSX2#-11, an Antarctic fungus derived from a soil sample of the Fields Peninsula, which can produce various compounds according to our previous study [13], was further investigated to search for new secondary metabolites. As a result, one new pyridine derivative, 4-(2-methoxycarbonyl-ethyl)-pyridine-2-carboxylic acid methyl ester (**1**), together with one known pyrimidine, thymine (**2**) and eight known diketopiperazines (**3**–**10**) (Figure 1), were isolated and identified from the potato dextrose agar (PDA) culture broth fermentative extracts of this strain. This paper addresses the isolation, structure elucidation, and bioactivity evaluation of the isolated compounds.

## 2. Results

**Figure 2:**
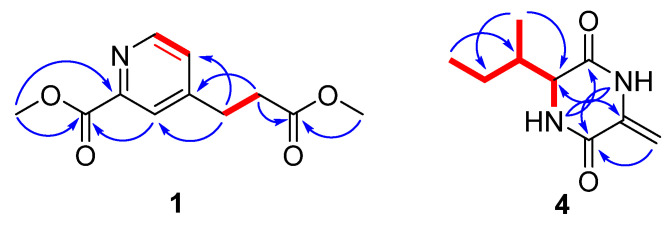
COSY (red bold line) and key HMBC (blue arrows) correlations of **1** and **4**.

4-(2-Methoxycarbonyl-ethyl)-pyridine-2-carboxylic acid methyl ester (**1**) was obtained as a colorless oil. The molecular formula of C_11_H_13_O_4_N was determined by high resolution electrospray ionization mass spectroscopy (HRESIMS) that displayed the [M + Na]^+^ peak at *m*/*z* 246.0742 (calcd for C_11_H_13_O_4_NNa, 246.0737), indicating six degrees of unsaturation (Figure S8 from Supplementary Materials). The ^1^H-NMR, ^13^C-NMR and heteronuclear single quantum coherence (HSQC) spectra (Figures S1, S2 and S6 in the Supplementary Materials) exhibited two methoxyls, (δ_H_ 3.66 (3H, s), δ_C_ 52.0; δ_H_ 3.98 (3H, s), δ_C_ 53.1), two methylenes, (δ_H_ 2.69 (2H, t, 7.5 Hz), δ_C_ 34.0; δ_H_ 3.03 (2H, t, 7.5 Hz), δ_C_ 30.1), three aromatic methines, (δ_H_ 7.37 (1H, br s), δ_C_ 127.4; δ_H_ 8.00 (1H, br s), δ_C_ 125.4; δ_H_ 8.66 (1H, br s), δ_C_ 149.6), two aromatic quaternary carbon signals (δ_C_ 147.6, δ_C_ 151.8), and two carbonyls (δ_C_ 165.4, δ_C_ 172.4) (Table 1). The five aromatic carbon signals combined with the molecular formula of C_11_H_13_O_4_N indicated a pyridine substructure in **1**. The splitting effects of ^1^H-NMR were undesirable in CDCl_3_ of the aromatic methines of **1**. So the ^1^H-NMR spectrum was measured again in DMSO-*d*_6_. The coupling constants of 5.0 Hz (H-5/H-6), 1.7 Hz (H-3/H-5) and 0.8 Hz (H-3/H-6) revealed the ortho-position of H-5/H-6, meta-position of H-3/H-5 and para-position of H-3/H-6, respectively (Table 1). The ^1^H-^1^H chemical-shift correlation spectroscopy (COSY) cross peak of H-9/H-10, and key heteronuclear multiple-bond correlation (HMBC) correlations of H-9/C-3, H-9/C-5, H-10/C-4, H-10/C-11 and H-12/C-11 elucidated the methyl propionate substituent located at C-4 (Figure 2). The methyl formate group at C-2 was suggested by the key HMBC correlations from H-8 to C-7 and C-2, and H-3 to C-7 (Figure 2). Thus compound **1** was identified as 4-(2-methoxycarbonyl-ethyl)-pyridine-2-carboxylic acid methyl ester.

*Cyclo*-(dehydroAla-l-Ile) (**4**) was obtained as a white powder. The HRESIMS spectrum of **4** led to the molecular formula of C_9_H_14_O_2_N_2_ (*m*/*z* 205.0975, calcd for C_9_H_14_O_2_N_2_Na, 205.0948), indicating 4 degrees of unsaturation (Figure S16 in the Supplementary Materials). The ^1^H-NMR, ^13^C-NMR and HSQC spectra (Figures S11, S12 and S14 in the Supplementary Materials) exhibited two methyls, (δ_H_ 0.85 (3H, t, 7.4 Hz), δ_C_ 11.7; δ_H_ 0.88 (3H, d, 7.0 Hz), δ_C_ 14.8), two methylenes, (δ_H_ 1.20–1.12, (1H, m), 1.41–1.33, (1H, m), δ_C_ 24.1; δ_H_ 4.76, (1H, br s), 5.17, (1H, br s), δ_C_ 98.8), two methines, (δ_H_ 1.85–1.80 (1H, m), δ_C_ 40.7; δ_H_ 3.91 (1H, t, 2.8), δ_C_ 59.6), one aromatic quaternary carbon signals (δ_C_ 134.7), and two carbonyls (δ_C_ 158.6, δ_C_ 165.4) (Table 1). The two NH signals (δ_H_ 8.34, (1H, br s), δ_H_ 10.53, (1H, s)) combined with two carbonyls indicated the diketopiperazine structure of **4**. The ^1^H-^1^H COSY correlations of H-5/H-7, H-7/H-10, H-7/H-8 and H-8/H-9 revealed the substitute group of 1-methyl-propyl. And the group was located at C-5 determined by the ^1^H-^1^H COSY cross peak of H-5 and N-4, and the HMBC correlation from H-10 to C-5. The dehydro-methyl substitute was located at C-2 by the HMBC signal from H-11 to C-2. Thus the planar structure of **4** was confirmed as *cyclo*-(dehydroAla-Ile) which was first isolated from marine bacteria *Claviceps purpurea* in 1994, while its detailed NMR data were not reported [14]. The absolute configuration of **4** was proposed to be same as **3** and **5** according to biogenetic perspective and their similar specific optical rotation (OR) data ([α${]}_{D}^{20}$ −19.3 (*c* 0.18, CH_3_OH) of **4** *vs* [α${]}_{D}^{20}$ −10.5 (*c* 0.18, CH_3_OH) of **3** and [α${]}_{D}^{20}$ −78.1 (*c* 0.18, CH_3_OH) of **5**, Table 2).

The structures of **2**, **3**, **5**–**10** were determined as thymine [15], *cyclo*-(dehydroAla-l-Val) [16,17], *cyclo*-(dehydroAla-l-Leu) [18], *cyclo*-(dehydroAla-l-Phe) [19], *cyclo*-(l-Val-l-Phe) [20], *cyclo*-(l-Leu-l-Phe) [20], *cyclo*-(l-Trp-l-Ile) [21] and *cyclo*-(l-Trp-l-Phe) [22], respectively, by comparing their NMR and specific OR data (Table 2) with those in the literature.

All the isolated compounds were evaluated for their antibacterial activities against a panel of bacteria, including four pathogenic bacteria, *E. coli*, *S. aureus*, *P. aeruginosa* and *B. subtilis*, and nine marine fouling bacteria, *P. fulva*, *A. hydrophila*, *A. salmonicida*, *V. anguillarum*, *V. harveyi*, *P. halotolerans*, *P. angustum*, *E. cloacae* and *E. hormaechei*, and cytotoxic activities against five human cancer cell lines A549, PANC-1, HCT116, HepG2 and MDA-MB-231. Unfortunately, none of the tested compounds showed any activity.

## 3. Materials and Methods

### 3.1. General Experimental Procedures

Optical rotations were measured on a JASCO P-1020 digital polarimeter (JASCO, Tokyo, Japan). UV spectra were recorded using an Implen Gmbh NanoPhotometer N50 Touch (Implen, Munich, Germany). NMR spectra were recorded on a Bruker AVANCE NEO (Bruker, Fällanden, Switzerland) at 600 MHz for ^1^H and 150 MHz for ^13^C in CDCl_3_ or DMSO-*d*_6_. Chemical shifts *δ* were recorded in ppm, using TMS as internal standard. HRESIMS spectra were measured on a Thermo Scientific LTQ Orbitrap XL spectrometer (Thermo Fisher Scientific, Bremen, Germany). HPLC separation was performed using a Hitachi Primaide Organizer Semi-HPLC system (Hitachi High Technologies, Tokyo, Japan) coupled with a Hitachi Primaide 1430 photodiodearray detector (Hitachi High Technologies). A Kromasil C_18_ semi-preparative HPLC column (250 × 10 mm, 5 µm) (Eka Nobel, Bohus, Sweden) was used. Silica gel (200–300 mesh; Qingdao Marine Chemical Group Co., Qingdao, China) and Sephadex LH-20 (Amersham Biosciences Inc., Piscataway, NJ, USA) were used for column chromatography. Precoated silica gel GF254 plates (Yantai Zifu Chemical Group Co., Yantai, China).

### 3.2. Fungal Materials

The fungus *Pseudogymnoascus* sp. HSX2#-11 was isolated from a soil sample of the Fields Peninsula at Chinese 35th Antarctic expedition in 2019. The strain was deposited in the State Key Laboratory of Microbial Technology, Institute of Microbial Technology, Shandong University, Qingdao, China, with the GenBank (NCBI) accession number MT367223.

### 3.3. Extraction and Isolation

The fungal strain *Pseudogymnoascus* sp. HSX2#-11 was fermented in a PDA liquid medium in 200 Erlenmeyer flasks (300 mL in each 1000 mL flask) at 16 °C for 45 days. The culture (60 L) was filtered to separate the broth from the mycelia. Then the mycelia were extracted three times with EtOAc (3 × 4000 mL) and then repeated extracted with CH_2_Cl_2_–MeOH (*v*/*v*, 1:1) three times (3 × 4000 mL). The broth was extracted repeatedly with EtOAc (3 × 60 L) to get the EtOAc layer. All the extracts were combined and were evaporated to dryness under reduced pressure to afford a residue (71.5 g). The residue was subjected to vacuum liquid chromatography (VLC) on silica gel using step gradient elution with EtOAc–petroleum ether (PE) (0–100%) and then with MeOH–EtOAc (0–100%) to afford eight fractions (Fr.1–Fr.8). Fr.3 was first subjected to gradient elution of octadecylsilyl silica gel (ODS) column chromatography (CC) with MeOH in H_2_O (10–100%), and then purified by using semi-preparative HPLC on an ODS column (Kromasil C_18_, 250 × 10 mm, 5 µm, 2 mL/min) eluted with 45% MeOH–H_2_O to give compounds **7** (5.5 mg), **8** (6.4 mg), **9** (3.5 mg) and **10** (4.4 mg). Fr.4 was isolated by CC on Sephadex LH-20 eluted with CH_2_Cl_2_–MeOH (*v*/*v*, 1:1) to afford two fractions (Fr.4.1, Fr.4.2). Fr.4.1 was first subjected to silica gel CC eluting with EtOAc–PE (0–50%), and then purified with HPLC eluted with 10% MeOH–H_2_O to give compound **2** (3.1 mg). Fr.7 was separated on CC on Sephadex LH-20 eluted with CH_2_Cl_2_–MeOH (*v*/*v*, 1:1) to afford three fractions (Fr.7.1–Fr.7.3). Fr. 7.2 was first subjected on HPLC eluted with 50% MeOH–H_2_O, and then purified with HPLC eluted with 20% MeOH–H_2_O to afford **3** (2.5 mg). Fr.7.3 was first isolated by HPLC eluted with 50% MeOH–H_2_O, and then purified with HPLC eluted with 30% MeOH–H_2_O to obtain **1** (2.1 mg), **4** (2.8 mg) and **5** (2.9 mg). Fr.8 was subjected to HPLC with 55% MeOH–H_2_O to gain **6** (7.6 mg).

4-(2-Methoxycarbonylethyl)-pyridine-2-carboxylic acid methyl ester (**1**): colorless oil; UV (MeOH) λ_max_ (log *ε*): 200 (4.70), 264 (4.11); ^1^H- and ^13^C-NMR data, see Table 1; HRESIMS *m*/*z* 246.0742 [M + Na]^+^ (calcd for C_11_H_13_O_4_NNa, 246.0737).

*Cyclo*-(dehydroAla-l-Ile) (**4**): white powder; [α${]}_{D}^{20}$ −19.3 (*c* 0.18, MeOH); UV (MeOH) λ_max_ (log *ε*): 247 (4.30), 288 (4.01); ^1^H- and ^13^C-NMR data, see Table 1; HRESIMS *m*/*z* 205.0975 [M + Na]^+^ (calcd for C_9_H_14_O_2_N_2_Na, 205.0948).

### 3.4. Antibacterial and Cytotoxic Activity Assays

The antibacterial activities were evaluated by the conventional broth dilution assay [23]. Four pathogenic bacteria, *Escherichia coli*, *Staphylococcus aureus*, *P. aeruginosa* and *Bacillus subtilis*, and nine marine fouling bacteria, *P. fulva*, *Aeromonas hydrophila*, *A. salmonicida*, *Vibrio anguillarum*, *V. harveyi*, *Photobacterium halotolerans*, *P. angustum*, *Enterobacter cloacae* and *E. hormaechei*, were used, and cipofloxacin was used as a positive control.

The cytotoxicities against human breast cancer (MDA-MB-231, Shanghai Institute of Biological Sciences, Chinese Academy of Sciences, L-15), colorectal cancer (HCT116, Shanghai Institute of Biological Sciences, Chinese Academy of Sciences, McCOY’s 5A), lung carcinoma (A549, Shanghai Institute of Biological Sciences, Chinese Academy of Sciences, F-12), pancreatic carcinoma (PANC-1, Shanghai Institute of Biological Sciences, Chinese Academy of Sciences, DMEM) and hepatoma (HepG2, Shanghai Institute of Biological Sciences, Chinese Academy of Sciences, DMEM) cell lines were evaluated using the sulphorodamine B (SRB) method [24]. Adriamycin was used as a positive control.

## 4. Conclusions

In summary, one new pyridine derivative, 4-(2-methoxycarbonyl-ethyl)-pyridine-2-carboxylic acid methyl ester (**1**), together with one pyrimidine, thymine (**2**), and eight diketopiperazines **3**–**10**, were isolated from the Antarctic fungus *Pseudogymnoascus* sp. HSX2#-11. All the isolated compounds showed no antibacterial or cytotoxic activities. More bioactivity evaluating models should be needed to find the effects of these secondary metabolites. This is the first time to find pyridine, pyrimidine and diketopiperazines from the genus of *Pseudogymnoascus*. Our chemical investigation of the Antarctic fungus *Pseudogymnoascus* sp. HSX2#-11 enriches the chemical diversity of this fungal species.

## Figures and Tables

**Table 1:** NMR spectroscopic data (600/150 MHz) for compounds **1** and **4**.

No.	1				4	
Pos	*δ*_C_^a^ Type	*δ*_H_^a^ Multiple (Hz)	*δ*_C_^b^ Type	*δ*_H_^b^ Multiple (Hz)	*δ*_C_^b^ Type	*δ*_H_^b^ Multiple (Hz)
1	-	-	-	-	-	10.53, s
2	147.6, C	-	147.5, C	-	134.7, C	-
3	125.4, CH	8.00, br s	124.9, CH	7.94, dd (1.7, 0.8)	158.6, C	-
4	151.8, C	-	151.1, C	-	-	8.34, br s
5	127.4, CH	7.37, br s	127.2, CH	7.52, dd (5.0, 1.7)	59.6, CH	3.91, t (2.8)
6	149.6, CH	8.66, br s	149.7, CH	8.59, dd (5.0, 0.8)	165.4, C	-
7	165.4, C	-	165.3, C	-	40.7, CH	1.85–1.80, m
8	53.1, CH_3_	3.98, s	52.4, CH_3_	3.87, s	24.1, CH_2_	1.41–1.33, m
	-	-	-	-	-	1.20–1.12, m
9	30.1, CH_2_	3.03, t (7.5)	29.2, CH_2_	2.95, t (7.5)	11.7, CH_3_	0.85, t (7.4)
10	34.0, CH_2_	2.69, t (7.5)	33.2, CH_2_	2.73, t (7.5)	14.8, CH_3_	0.88, d (7.0)
11	172.4, C	-	172.3, C	-	98.8, CH_2_	5.17, br s
	-	-	-	-	-	4.76, br s
12	52.0, CH_3_	3.66, s	51.4, CH_3_	3.58, s	-	-

^a^ measured in CDCl_3_, ^b^ measured in DMSO-*d*_6_.

**Table 2:** Specific OR of diketopiperazines **3**–**10** in CH_3_OH.

Compounds	Natural [α]${}_{D}^{20}$	Literature
3	−10.5 (*c* 0.18)	[α${]}_{D}^{26}$ −95.7 (*c* 0.4) [17]
4	−19.3 (*c* 0.18)	-
5	−78.1 (*c* 0.18)	[α${]}_{D}^{22}$ −163 (0.01) [18]
6	−41.3 (*c* 0.18)	[α${]}_{D}^{26}$ −40.0 (1.08) ^a^ [19]
7	−25.3 (*c* 0.18)	[α${]}_{D}^{25}$ −11.4 (1.0) ^a^ [20]
8	+18.9 (*c* 0.14)	[α${]}_{D}^{25}$ +30.0 (0.3) [20]
9	−16.5 (*c* 0.18)	[α${]}_{D}^{25}$ −31.5 (0.0065) [21]
10	−144.2 (*c* 0.14)	[α${]}_{D}^{20}$ −254.9 (1.0) [22]

^a^ measured in DMSO.

## Data Availability

The fungus *Pseudogymnoascus* sp. HSX2#-11’s ribosomal RNA gene, partial sequence can be found at https://www.ncbi.nlm.nih.gov/nuccore/MT367223.1/.

## Supplementary Materials

The following are available online, NMR and HRESIMS spectra of the isolated compounds **1**–**10**: Figure S1: ^1^H NMR spectrum of compound **1** (CDCl_3_); Figure S2: ^13^C NMR spectrum of compound **1** (CDCl_3_); Figure S3: ^1^H NMR spectrum of compound **1** (DMSO-*d*_6_); Figure S4: ^13^C NMR spectrum of compound **1** (DMSO-*d*_6_); Figure S5: COSY spectrum of compound **1** (CDCl_3_); Figure S6: HSQC spectrum of compound **1** (CDCl_3_); Figure S7: HMBC spectrum of compound **1** (CDCl_3_); Figure S8: HRESIMS spectrum of compound **1**; Figure S9: ^1^H NMR spectrum of compound **2** (DMSO-*d*_6_); Figure S10: ^1^H NMR spectrum of compound **3** (DMSO-*d*_6_); Figure S11: ^1^H NMR spectrum of compound **4** (DMSO-*d*_6_); Figure S12: ^13^C NMR spectrum of compound **4** (DMSO-*d*_6_); Figure S13: COSY spectrum of compound **4** (DMSO-*d*_6_); Figure S14: HSQC spectrum of compound **4** (DMSO-*d*_6_); Figure S15: HMBC spectrum of compound **4** (DMSO-*d*_6_); Figure S16: HRESIMS spectrum of compound **4**; Figure S17: ^1^H NMR spectrum of compound **5** (DMSO-*d*_6_); Figure S18: ^1^H NMR spectrum of compound **6** (DMSO-*d*_6_); Figure S19: ^1^H NMR spectrum of compound **7** (DMSO-*d*_6_); Figure S20: ^1^H NMR spectrum of compound **8** (DMSO-*d*_6_); Figure S21: ^1^H NMR spectrum of compound **9** (DMSO-*d*_6_); Figure S22: ^1^H NMR spectrum of compound **10** (DMSO-*d*_6_).

## Sample Availability

Samples of the compounds **1**–**10** are available from the authors.
