# Supplementary material for: Nitrogenous Compounds from the Antarctic Fungus Pseudogymnoascus sp. HSX2#-11
Source: Molecules. 2021 Apr 30;26(9):2636. doi: 10.3390/molecules26092636 (PMC8124204; doi:10.3390/molecules26092636)
Supplement: Supplementary file 1 [file molecules-26-02636-s001.zip › molecules-1174616-supplementary.pdf]

Nitrogenous compounds from the Antarctic fungus *Pseudogymnoascus* sp. HSX2#-11

Ting Shi, Li Zheng, Xiang-Qian Li, Jia-Jia Dai, Yi-Ting Zhang, Yan-Yan Yu, Wen-Peng Hu and Da-Yong Shi*

Figure S1. ^{1}H NMR spectrum of compound **1** ($CDCl_3$).
Figure S2. ^{13}C NMR spectrum of compound **1** ($CDCl_3$).
Figure S3. ^{1}H NMR spectrum of compound **1** (DMSO-d_6).
Figure S4. ^{13}C NMR spectrum of compound **1** (DMSO-d_6).
Figure S5. COSY spectrum of compound **1** ($CDCl_3$).
Figure S6. HSQC spectrum of compound **1** ($CDCl_3$).
Figure S7. HMBC spectrum of compound **1** ($CDCl_3$).
Figure S8. HRESIMS spectrum of compound **1**.
Figure S9. ^{1}H NMR spectrum of compound **2** (DMSO-d_6).
Figure S10. ^{1}H NMR spectrum of compound **3** (DMSO-d_6).
Figure S11. ^{1}H NMR spectrum of compound **4** (DMSO-d_6).
Figure S12. ^{13}C NMR spectrum of compound **4** (DMSO-d_6).
Figure S13. COSY spectrum of compound **4** (DMSO-d_6).
Figure S14. HSQC spectrum of compound **4** (DMSO-d_6).
Figure S15. HMBC spectrum of compound **4** (DMSO-d_6).
Figure S16. HRESIMS spectrum of compound **4**.
Figure S17. ^{1}H NMR spectrum of compound **5** (DMSO-d_6).
Figure S18. ^{1}H NMR spectrum of compound **6** (DMSO-d_6).
Figure S19. ^{1}H NMR spectrum of compound **7** (DMSO-d_6).
Figure S20. ^{1}H NMR spectrum of compound **8** (DMSO-d_6).
Figure S21. ^{1}H NMR spectrum of compound **9** (DMSO-d_6).
Figure S22. ^{1}H NMR spectrum of compound **10** (DMSO-d_6).

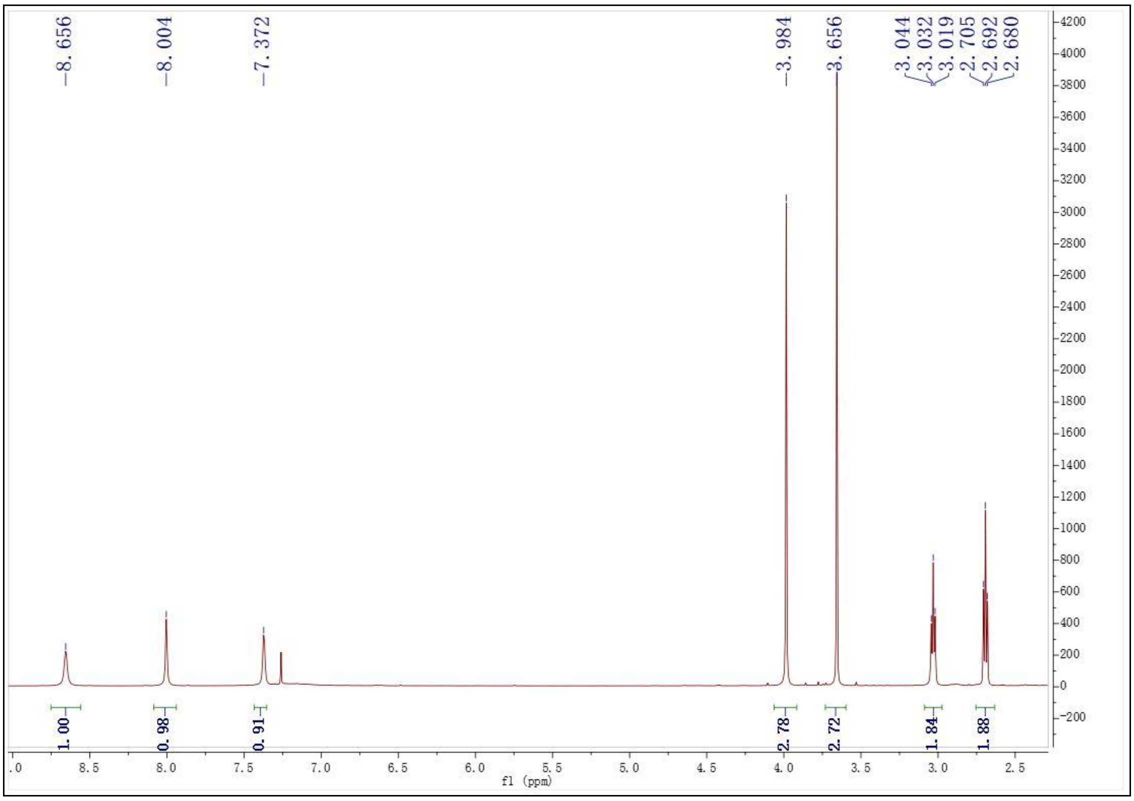

Figure S1. ^{1}H NMR spectrum of compound **1** ($CDCl_3$).

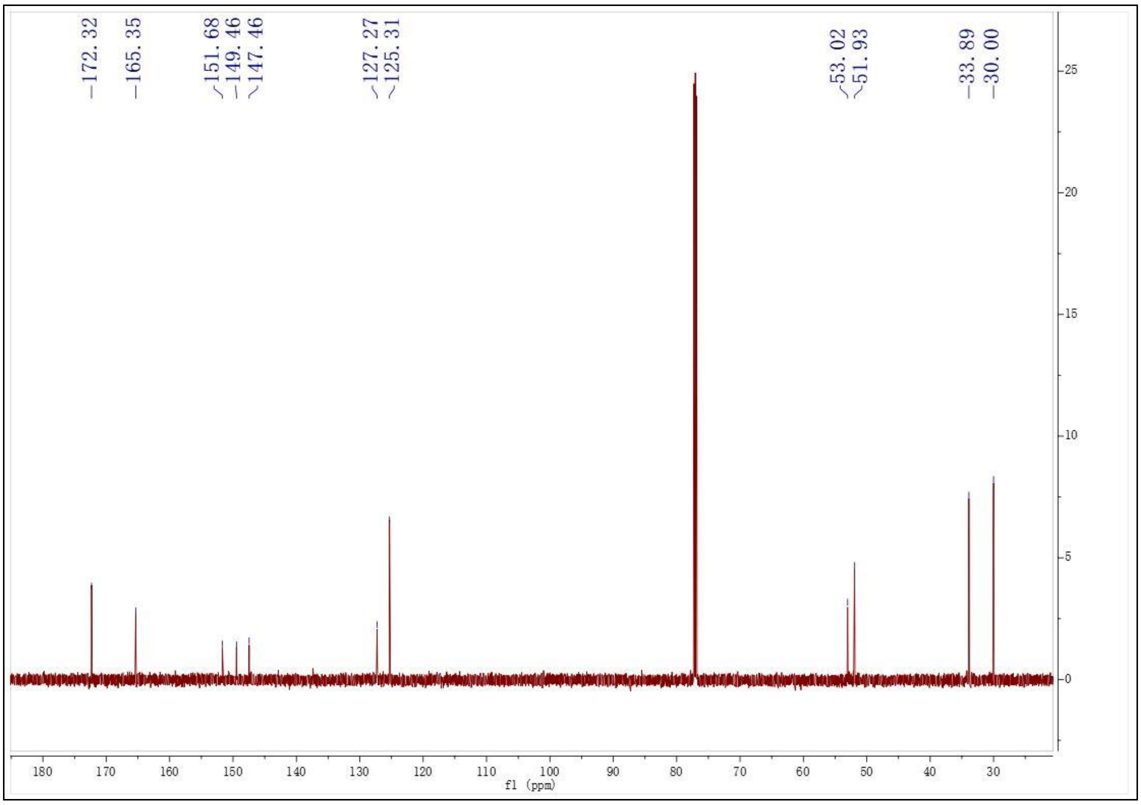

Figure S2. ^{13}C NMR spectrum of compound **1** ($CDCl_3$).

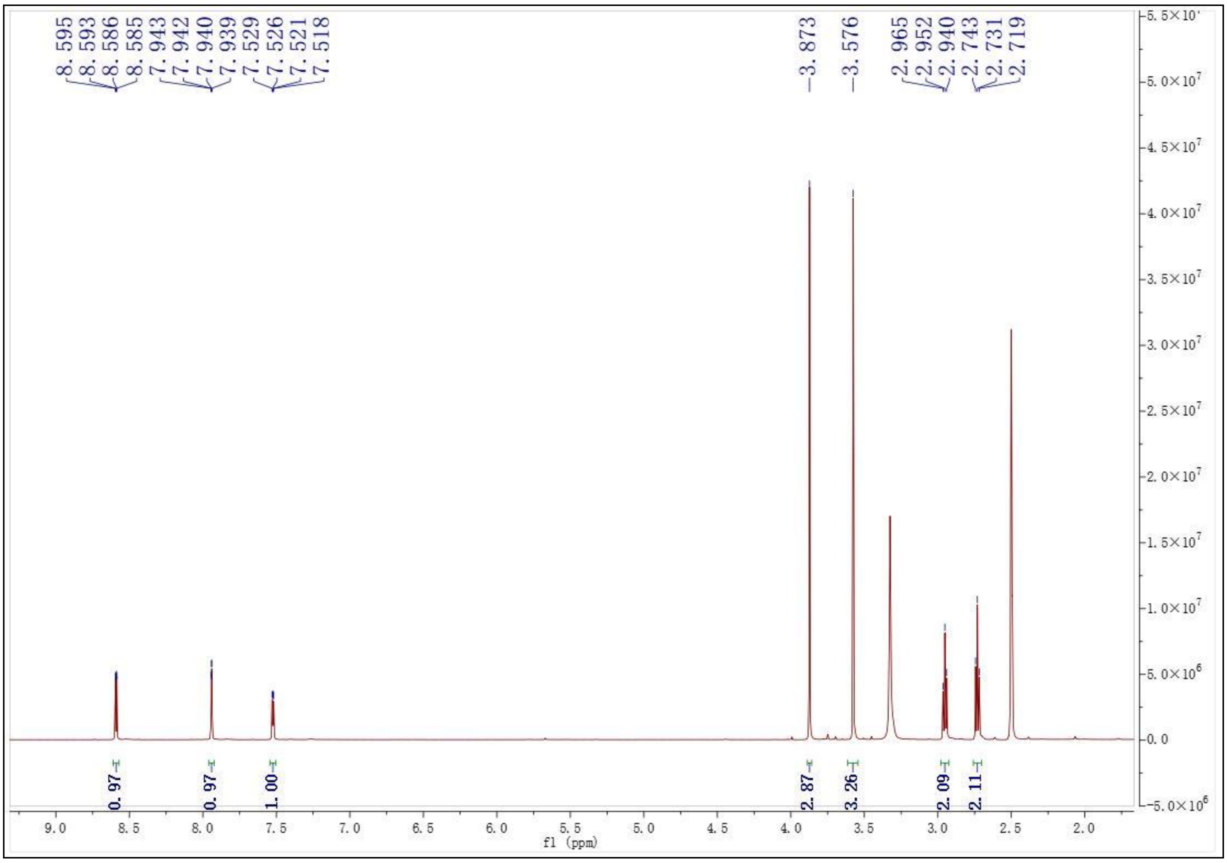

Figure S3. ^{1}H NMR spectrum of compound **1** (DMSO-d_6).

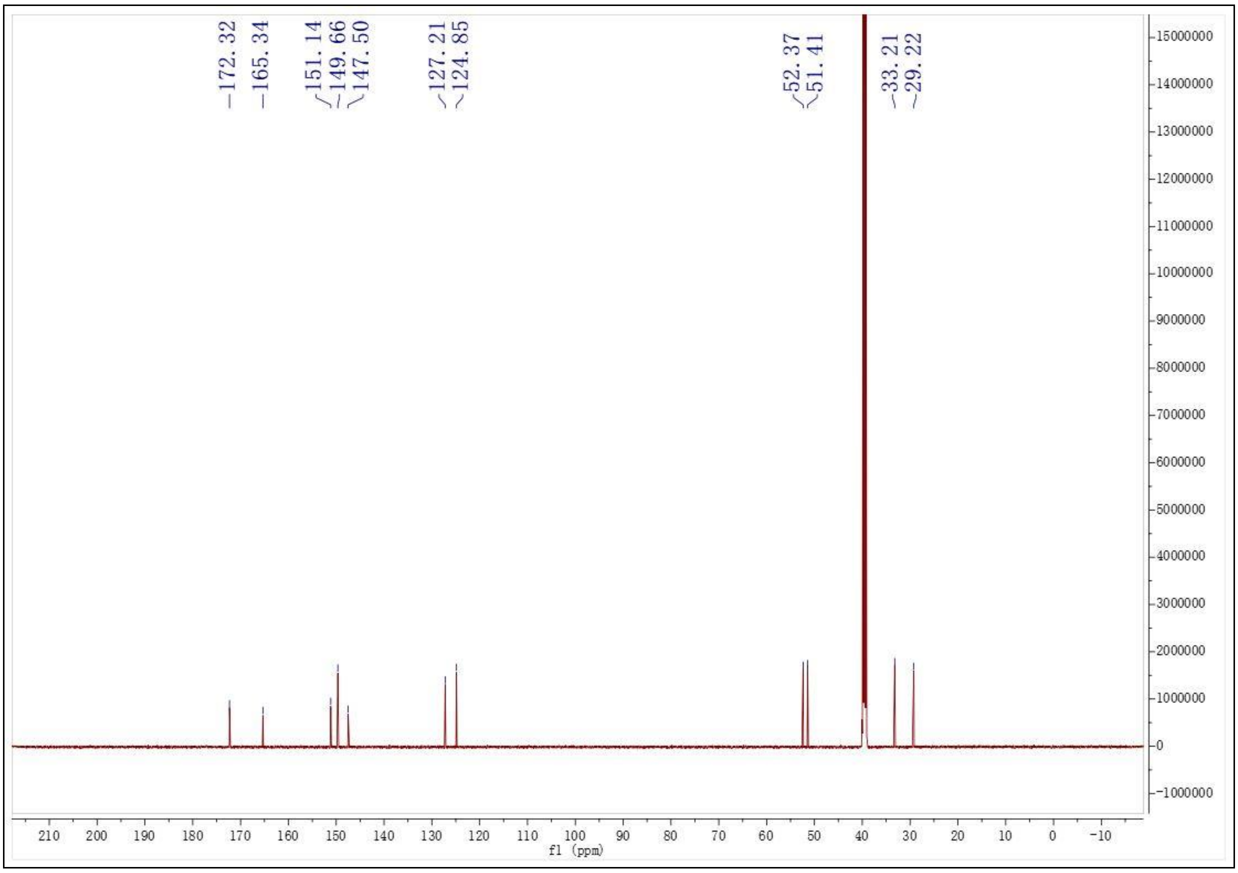

Figure S4. ^{13}C NMR spectrum of compound **1** (DMSO-d_6).

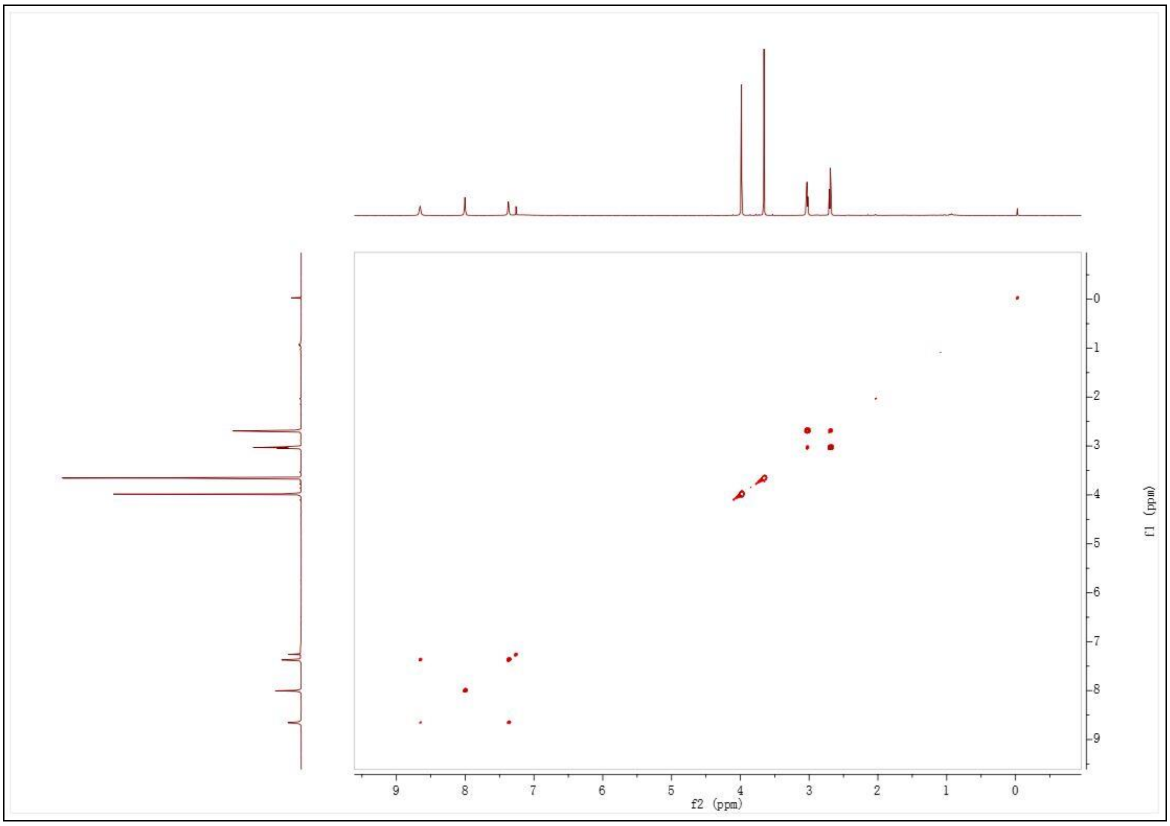

Figure S5. COSY spectrum of compound **1** ($CDCl_3$).

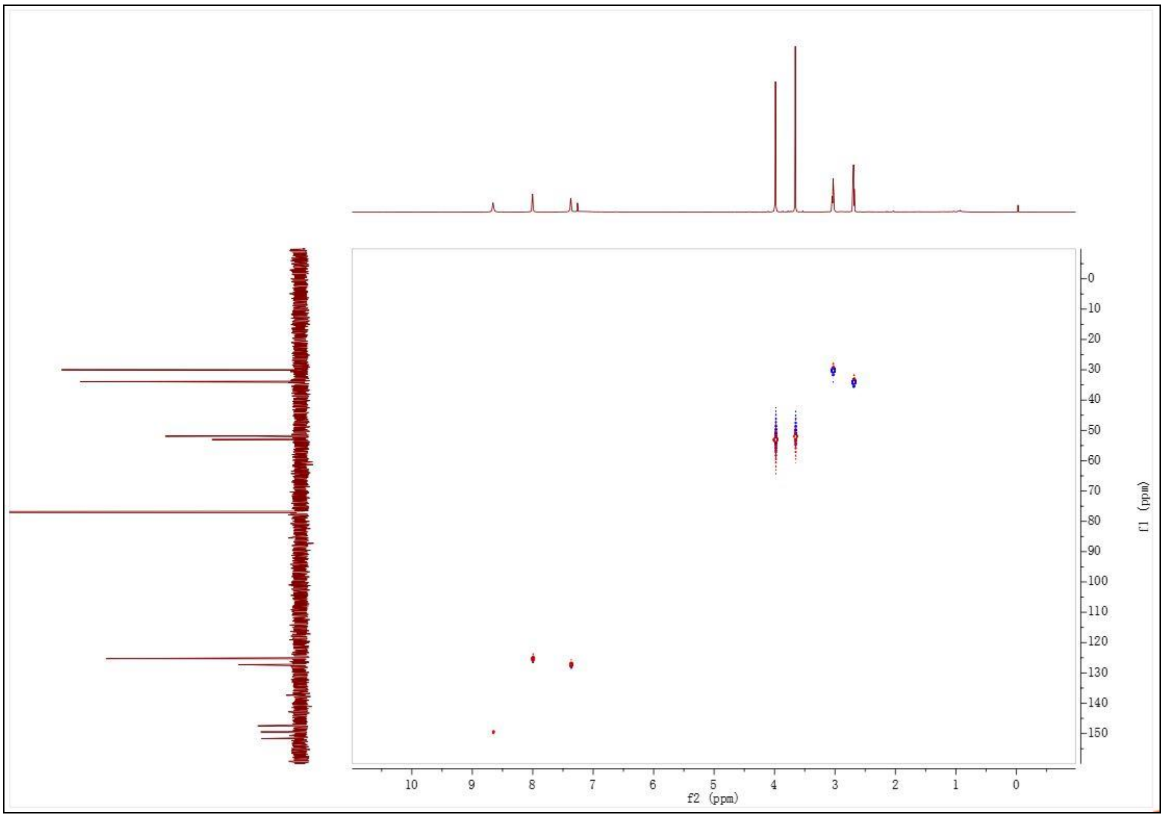

Figure S6. HSQC spectrum of compound **1** ($CDCl_3$).

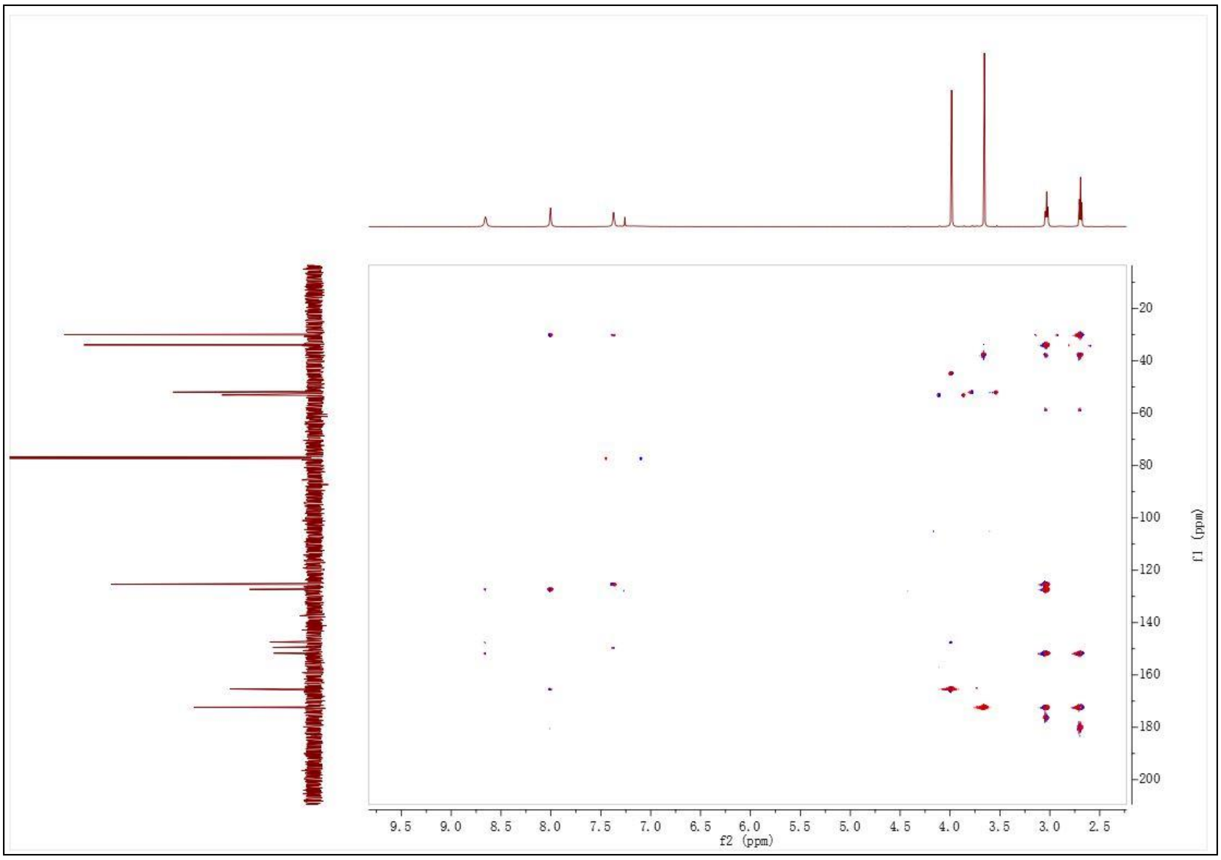

Figure S7. HMBC spectrum of compound **1** ($CDCl_3$).

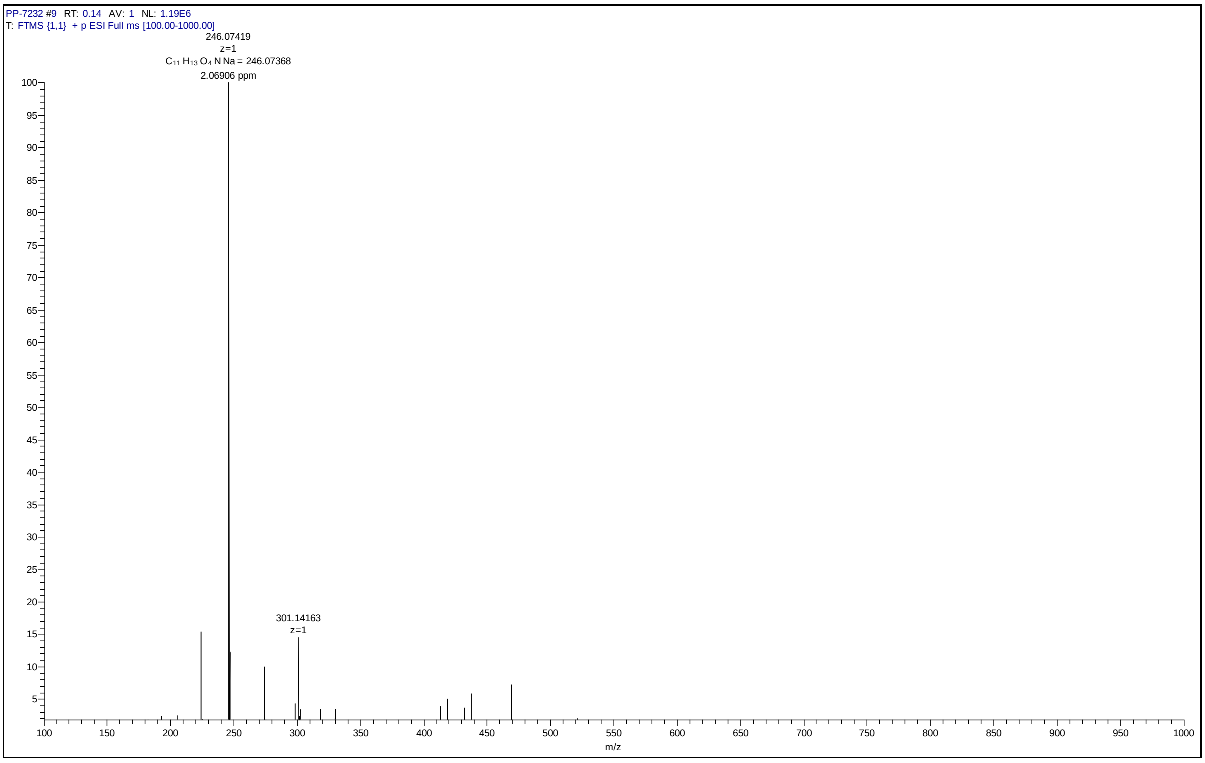

Figure S8. HRESIMS spectrum of compound **1**.

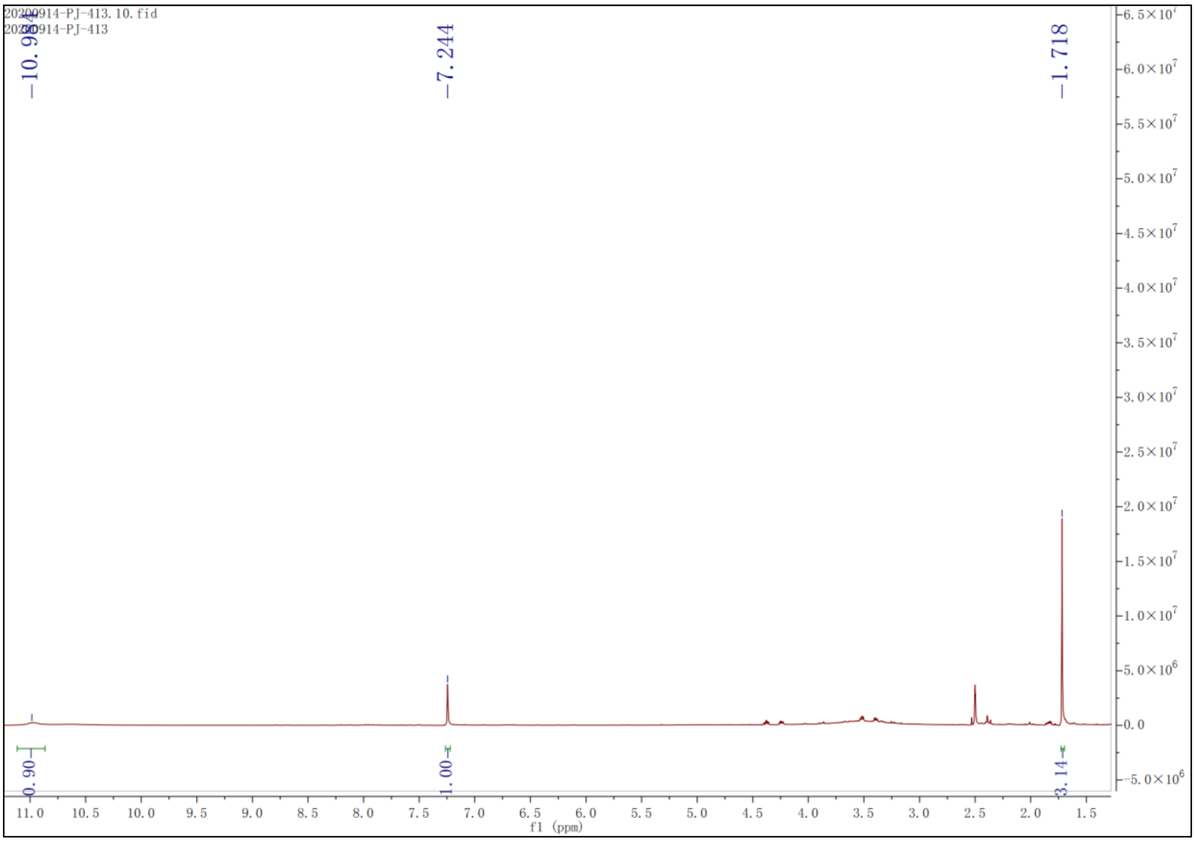

Figure S9. ^{1}H NMR spectrum of compound **2** (DMSO-d_6).

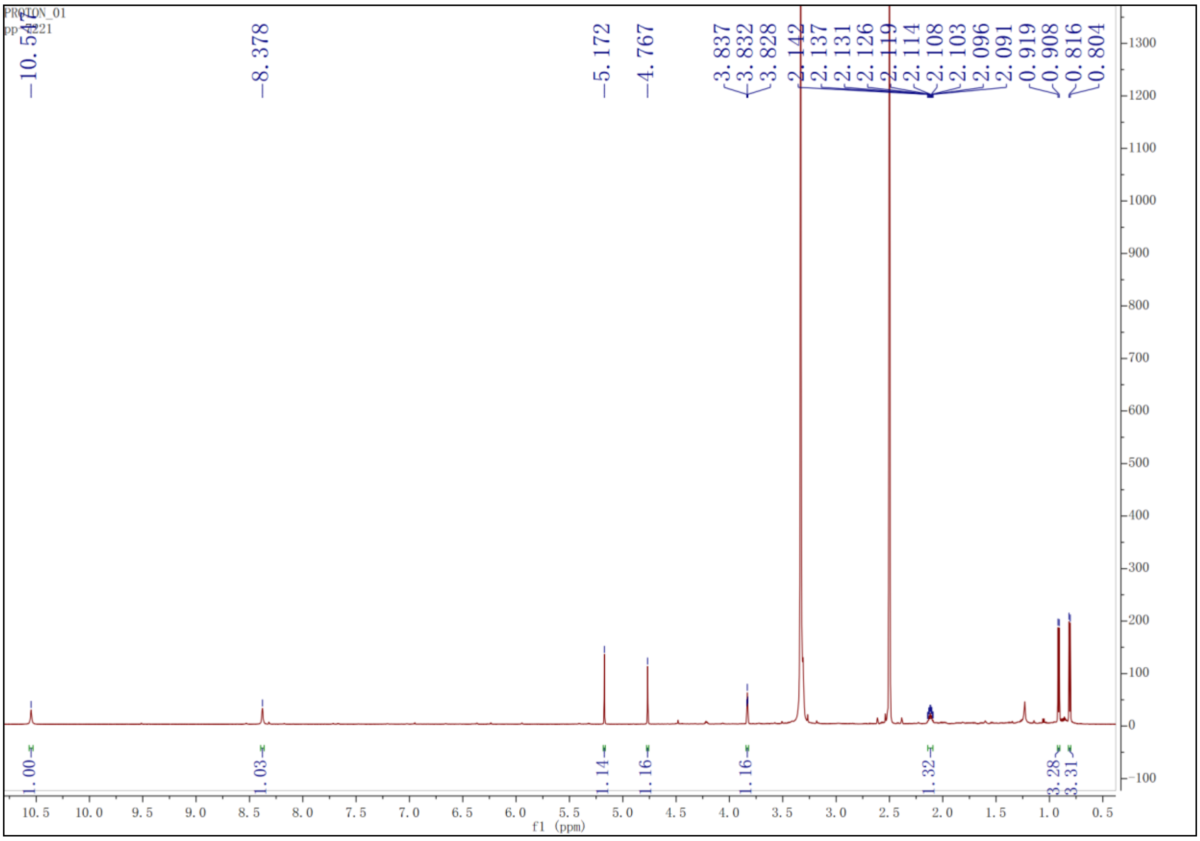

Figure S10. ^{1}H NMR spectrum of compound **3** (DMSO-d_6).

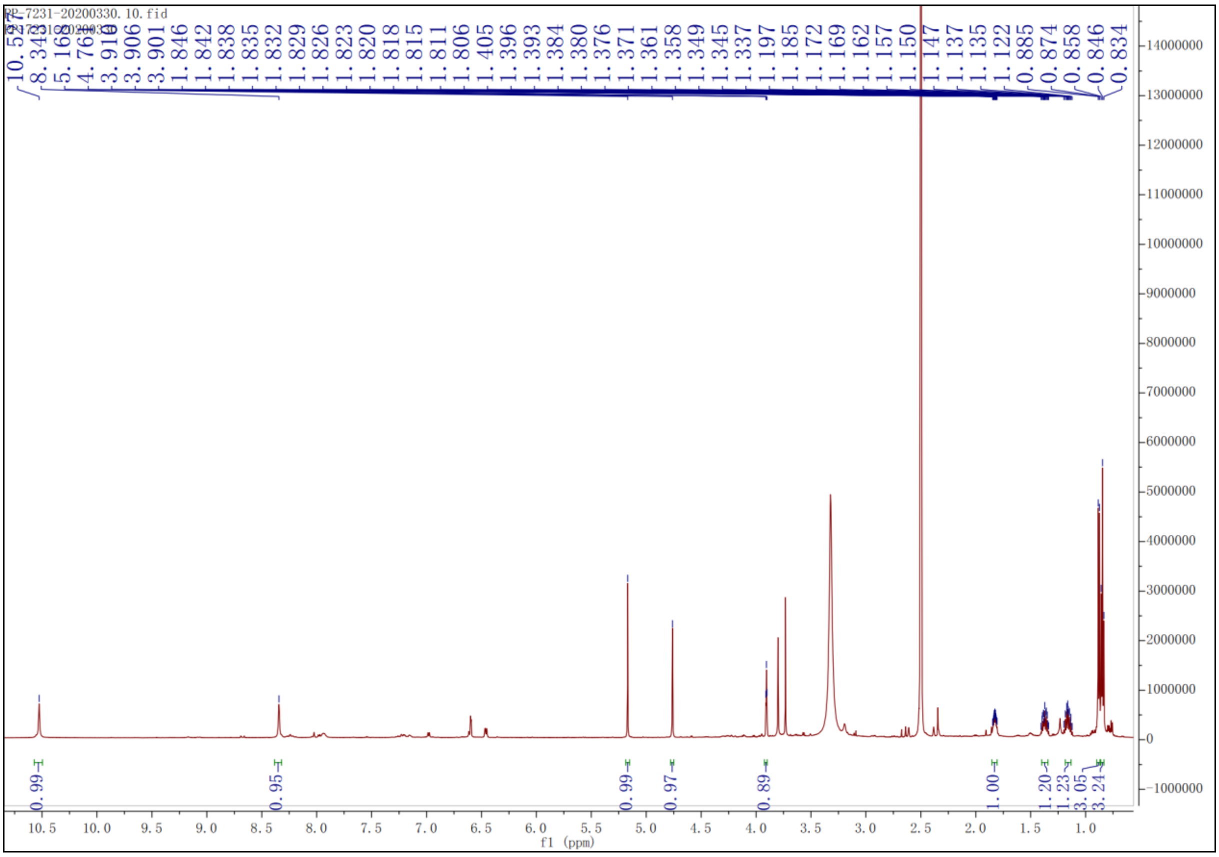

Figure S11. ^{1}H NMR spectrum of compound **4** (DMSO-d_6).

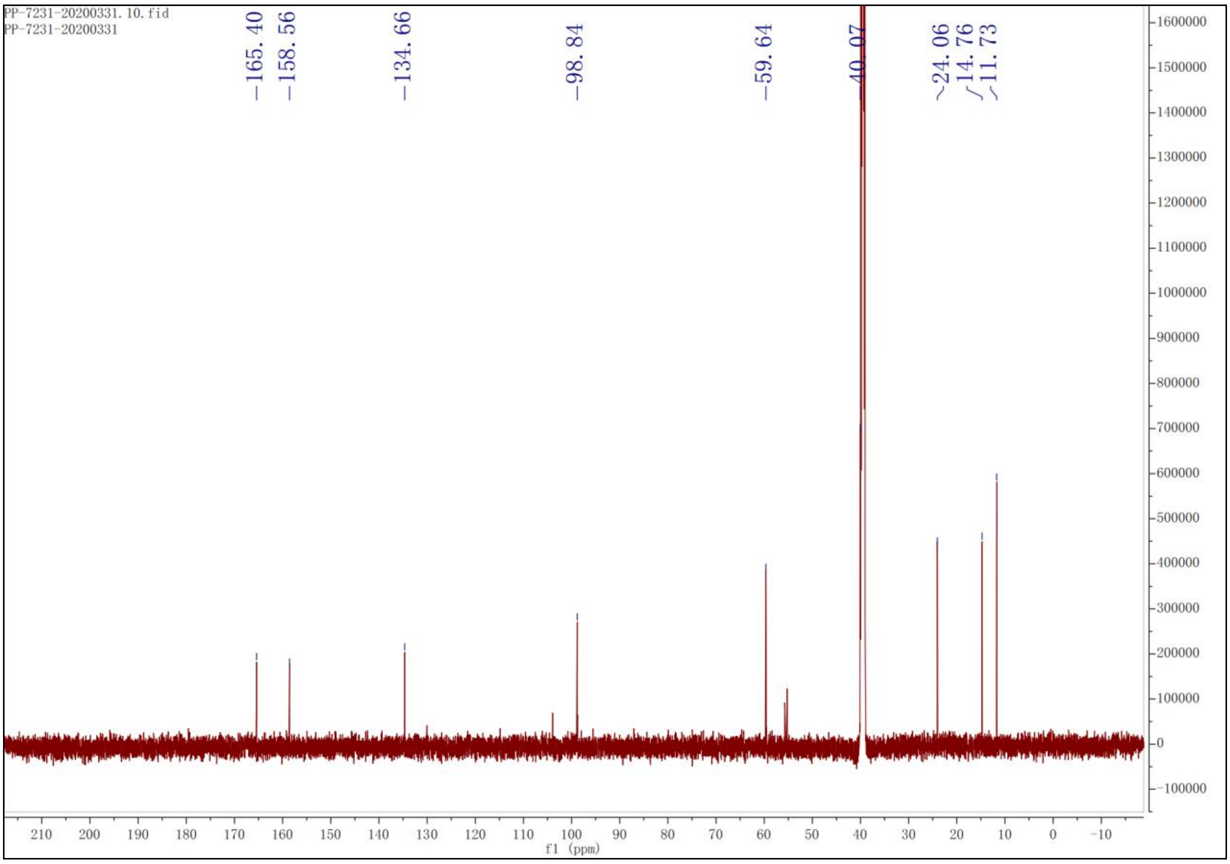

Figure S12. ^{13}C NMR spectrum of compound **4** (DMSO-d_6).

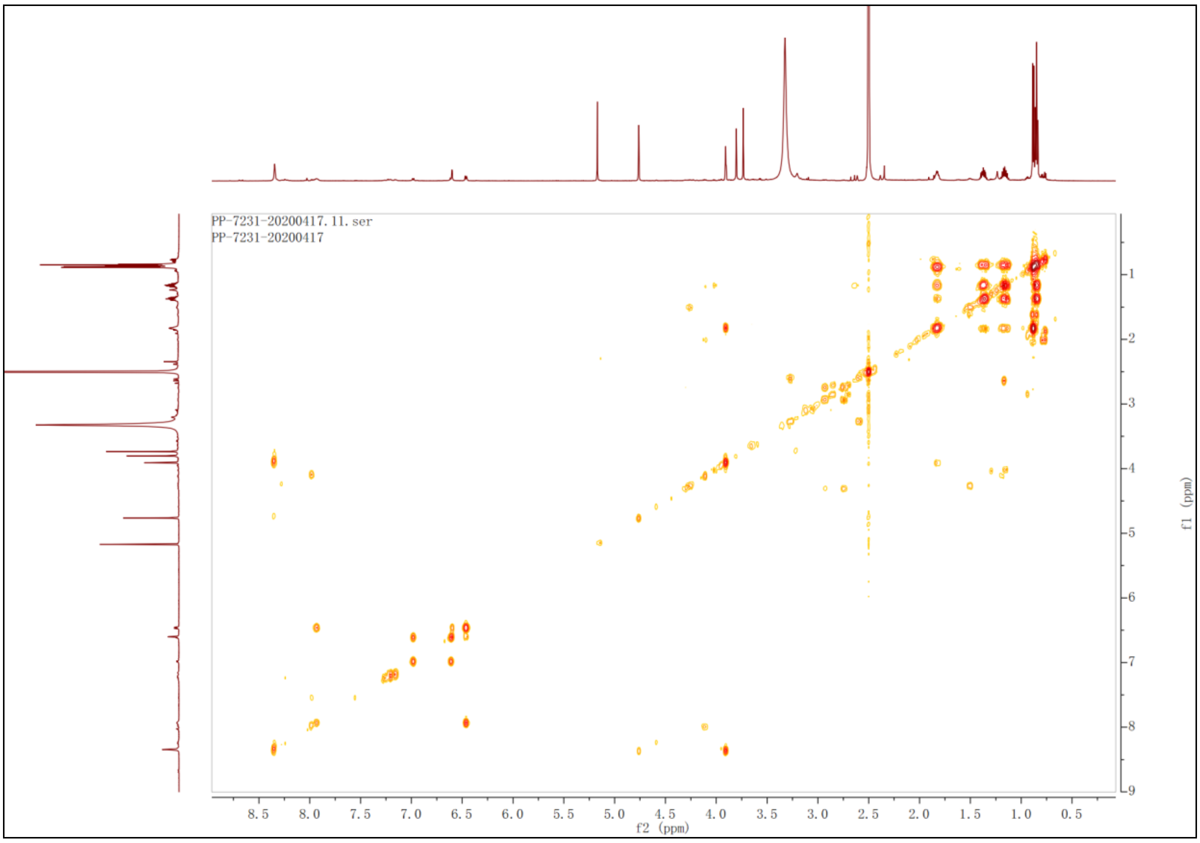

Figure S13. COSY spectrum of compound **4** (DMSO-d_6).

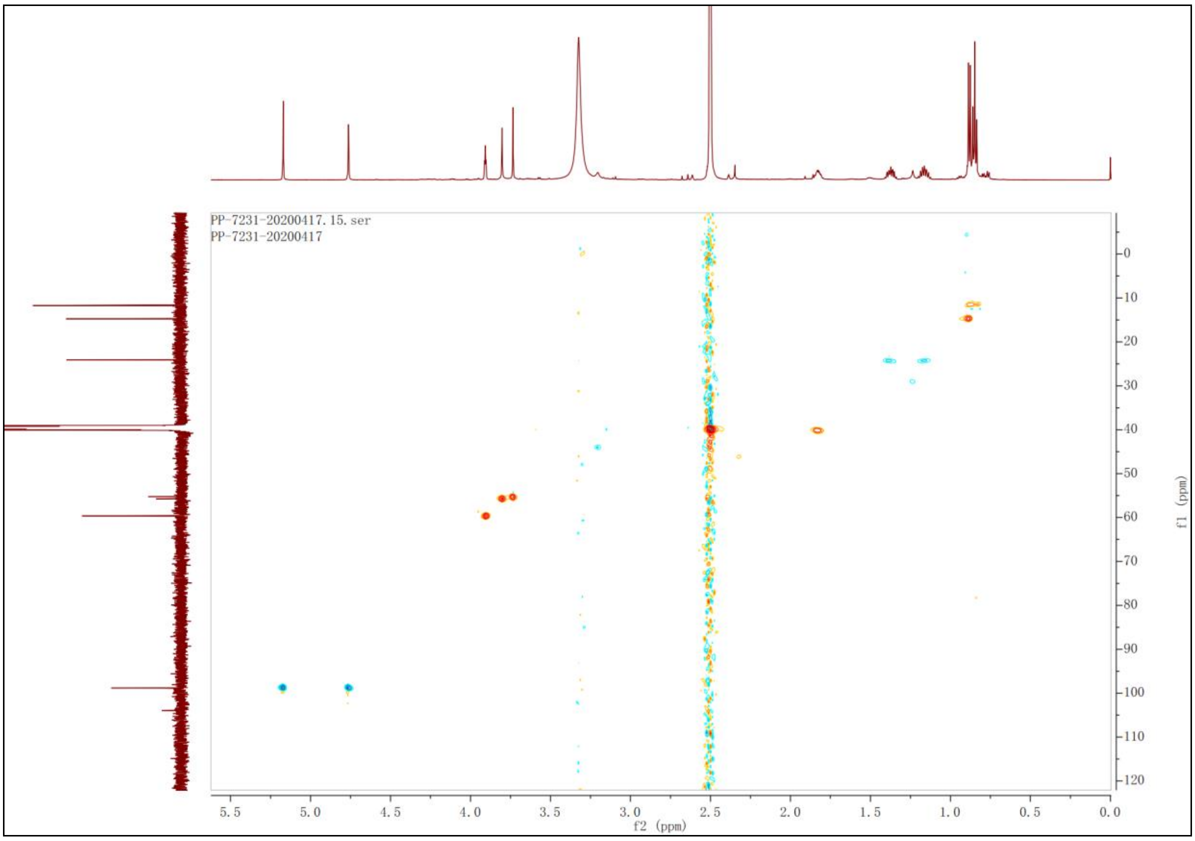

Figure S14. HSQC spectrum of compound **4** (DMSO-d_6).

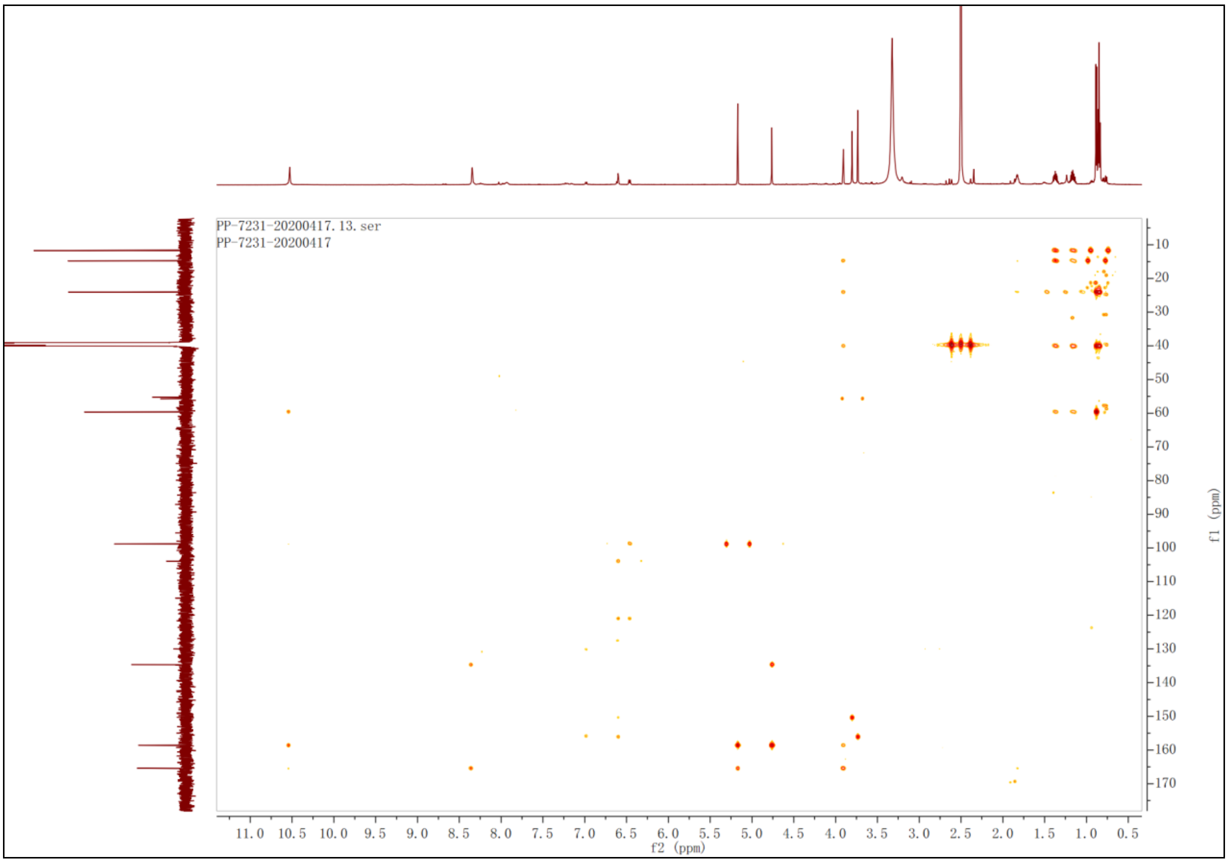

Figure S15. HMBC spectrum of compound **4** (DMSO-d_6).

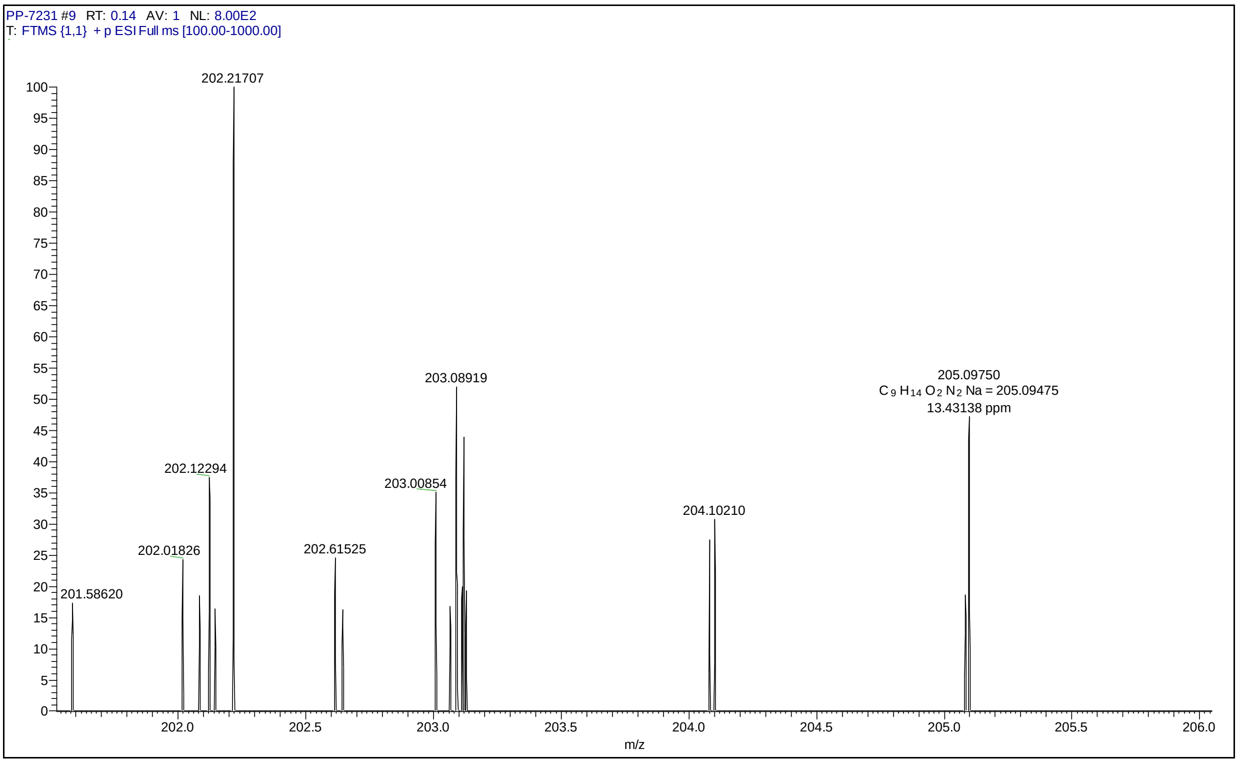

Figure S16. HRESIMS spectrum of compound **4**.

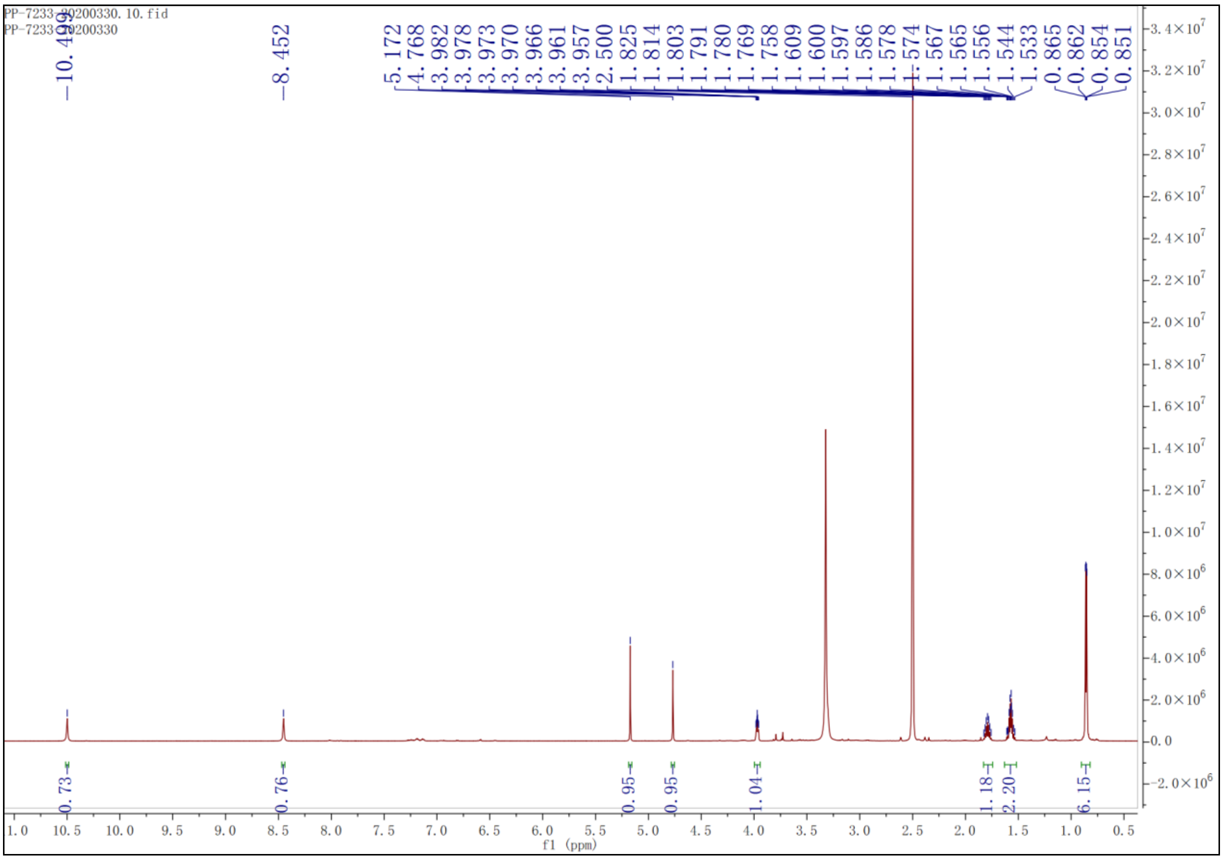

Figure S17. ^{1}H NMR spectrum of compound **5** (DMSO-d_6).

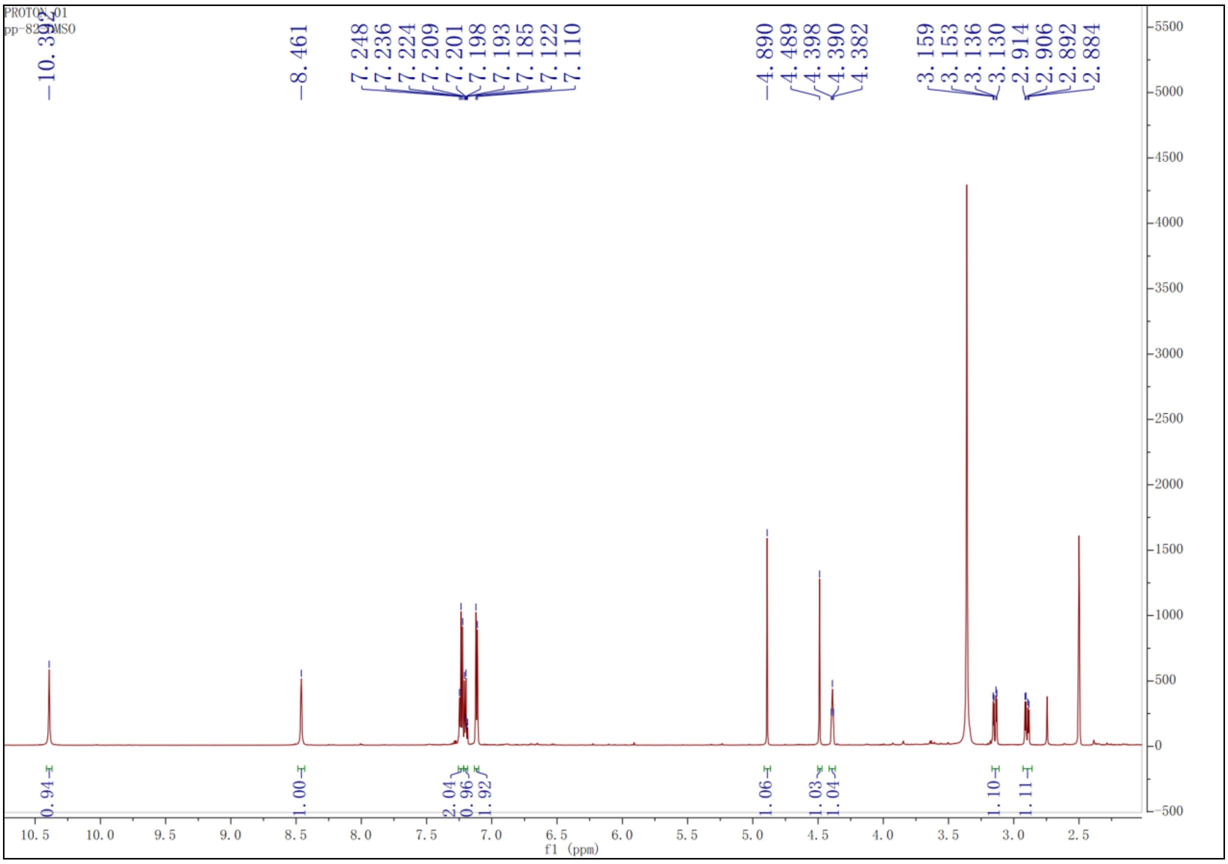

Figure S18. ^{1}H NMR spectrum of compound **6** (DMSO-d_6).

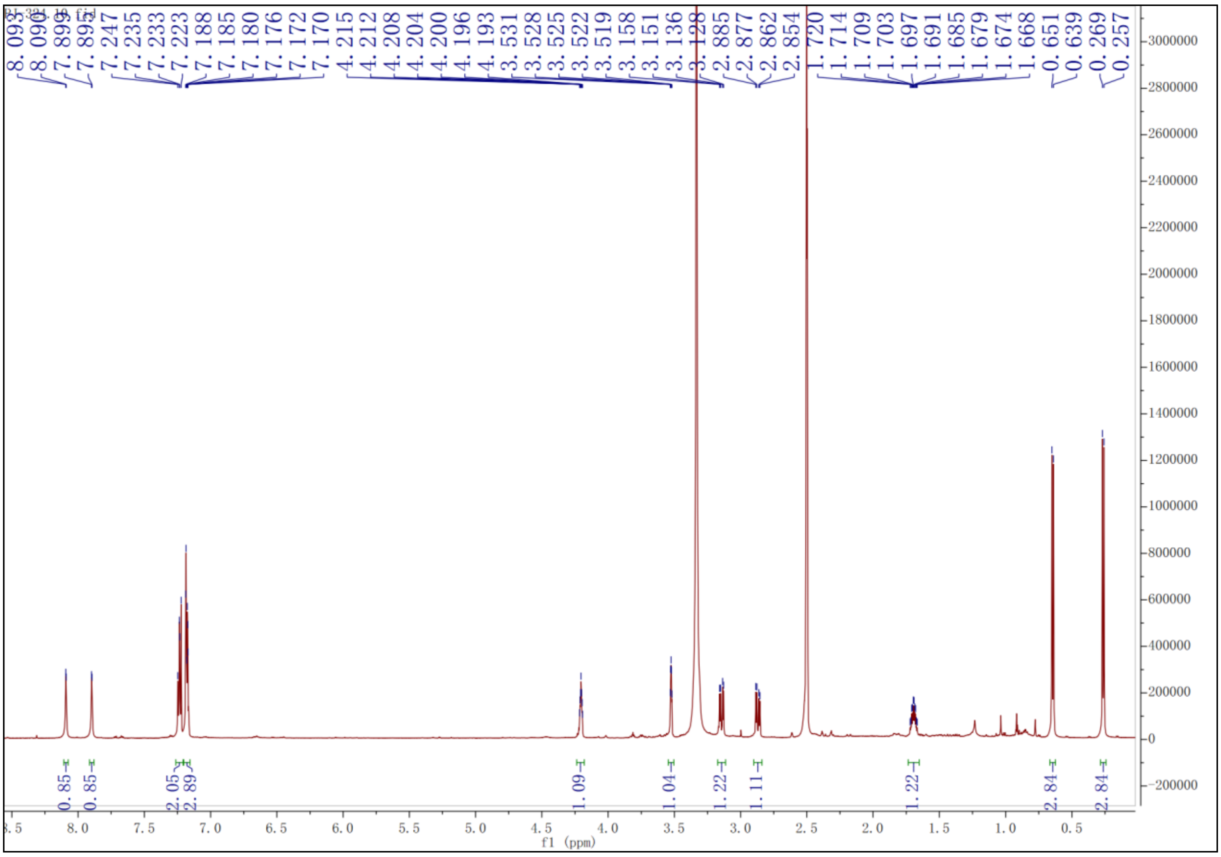

Figure S19. ^{1}H NMR spectrum of compound **7** (DMSO-d_6).

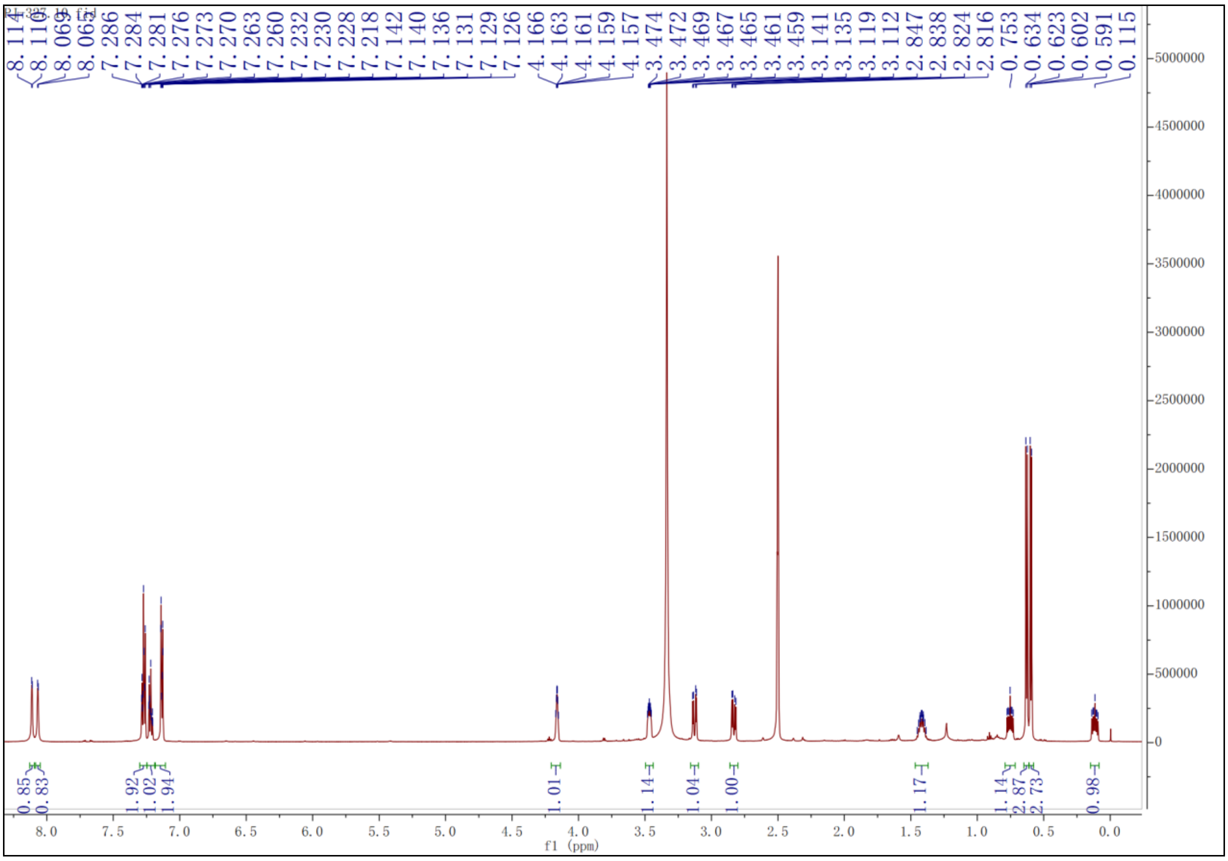

Figure S20. ^{1}H NMR spectrum of compound **8** (DMSO-d_6).

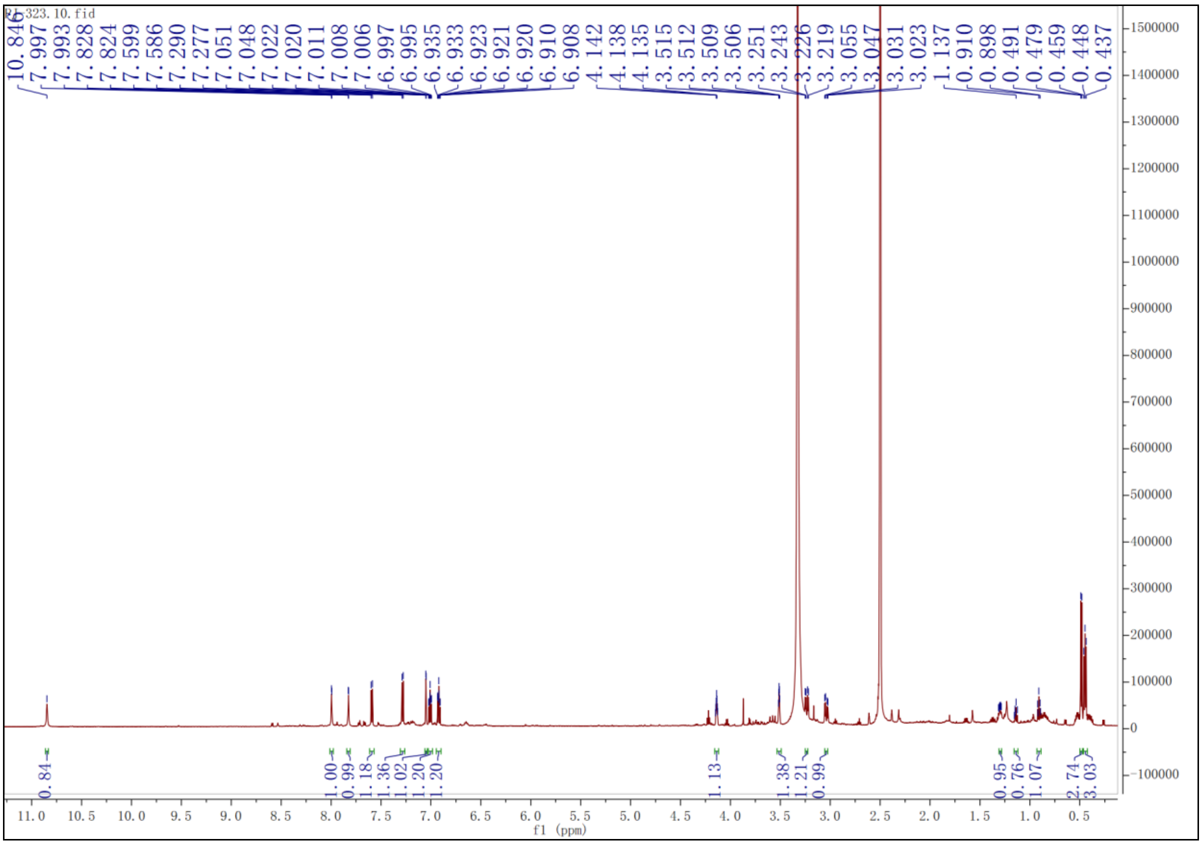

Figure S21. ^{1}H NMR spectrum of compound **9** (DMSO-d_6).

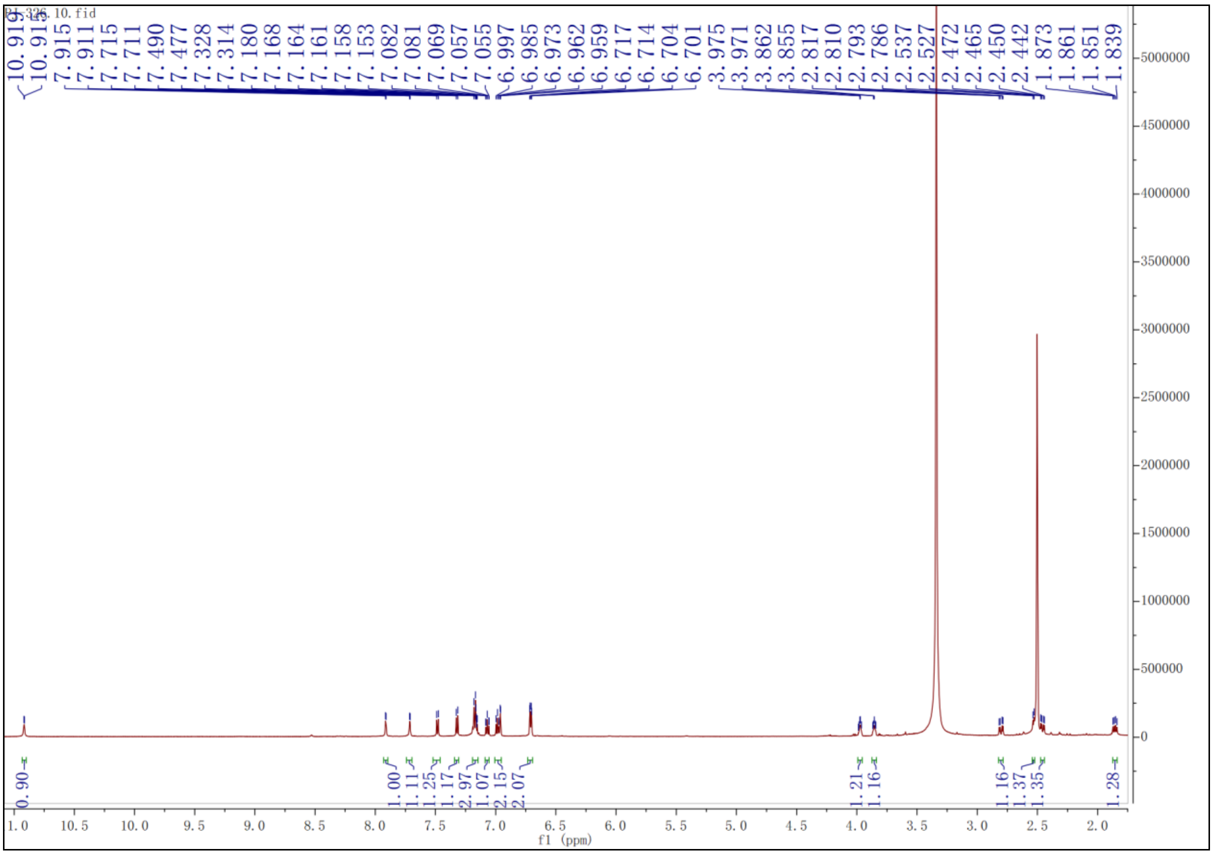

Figure S22. ^{1}H NMR spectrum of compound **10** (DMSO-d_6).
